# Tunable topological electronic states in the honeycomb-kagome lattices of nitrogen/oxygen-doped graphene nanomeshes†

**DOI:** 10.1039/d2na00132b

**Published:** 2022-04-06

**Authors:** Yiming Lu, Xuejia Fan, Xikui Ma, Jian Liu, Yangyang Li, Mingwen Zhao

**Affiliations:** School of Physics, Shandong University Jinan Shandong 250100 China mxk2022@sdu.edu.cn zmw@sdu.edu.cn

## Abstract

The rich and exotic electronic properties of graphene nanomeshes (GNMs) have been attracting interest due to their superiority to pristine graphene. Using first-principles calculations, we considered three graphene meshes doped with nitrogen and oxygen atoms (C_10_N_3_, C_9_N_4_ and C_10_O_3_). The electronic band structures of these GNMs in terms of the proximity of the Fermi level featured a two-dimensional (2D) honeycomb-kagome lattice with concurrent kagome and Dirac bands. The position of the Fermi level can be regulated by the doping ratio, resulting in different topological quantum states, namely topological Dirac semimetals and Dirac nodal line (DNL) semimetals. More interestingly, the adsorption of rhenium (Re) atoms in the voids of the C_10_N_3_ (Re@ C_10_N_3_) GNMs induced quantum anomalous Hall (QAH) states, as verified by the nonzero Chern numbers and chiral edge states. These GNMs offer a promising platform superior to pristine graphene for regulating multiple topological states.

## Abstract 2

The rich and exotic electronic properties of graphene nanomeshes (GNMs) have been attracting interest due to their superiority to pristine graphene.

## Graphical abstract

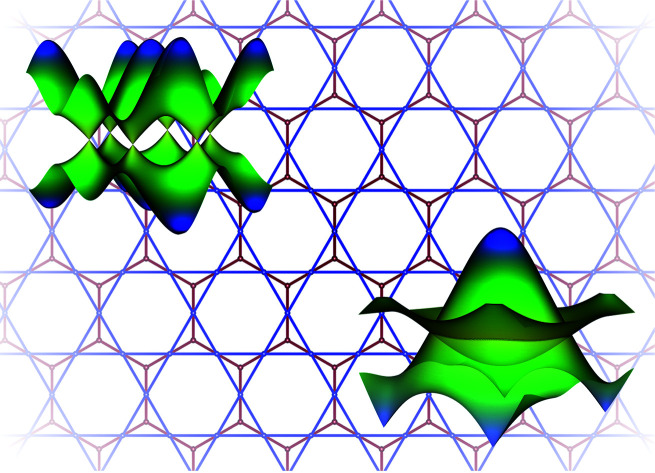

## Introduction

The discovery of topological insulators (TIs)^1–4^ has aroused extensive investigation into topologically nontrivial phases in quantum materials, bringing about new concepts for next-generation devices. The topological phases are closely correlated to the symmetries of materials, such as time-reversal symmetry, particle–hole symmetry, chirality and the lattice symmetries. TIs exhibit topologically protected conductive edge states on their surfaces or boundaries due to the spin-orbit coupling effect (SOC). In terms of two-dimensional (2D) topological insulators, the back scattering of conductive electrons on the one-dimensional (1D) boundary is completely forbidden, which allow electrons to be conducted loss-free at the boundaries of 2D TIs, which are then immune to impurity scattering and other disturbances. Therefore, 2D TIs are quite promising for low-energy electronics and spintronics. The incorporation of magnetic atoms into TIs can break the time-reversal symmetry, leaving conductive electrons with a particular spin transport on the boundary and realizing the quantum anomalous Hall effect (QAHE).^5–8^ One promising approach to achieve this strategy is to deposit transition metal (TM) atoms onto the surface of TIs, *e.g.* 5d TM metals onto graphene.^9–13^ However, the TM metal atoms deposited on graphene have a strong clustering tendency due to the relatively weak anchoring ability of graphene to the TM atoms, which makes the topological states difficult to achieve in experiments.^14^

In contrast to normal metals, topological semimetals have an intersection of their valence and conduction bands accompanied by a conical linear energy–momentum dispersion relationship.^15,16^ According to the modes of band crossing, topological semimetals can be classified into three types: topological Dirac semimetals,^17^ topological Weyl semimetals^18^ and Dirac nodal line (DNL) semimetals.^19–25^ For Dirac semimetals and Weyl semimetals, the valence band and the conduction band intersect at isolated points, which are quadruple degenerate and double degenerate, respectively, while DNL semimetals have a continuous closed curve intersection of their valence and conduction bands in the momentum space. The linear dispersion relation at the Fermi level of topological semimetals contributes to many exotic properties, such as ultra-high carrier mobility,^26^ high-temperature linear quantum reluctance,^27,28^ oscillating quantum spin Hall effect^29^ and giant reluctance effect.^30,31^

Motivated by these intriguing properties, a series of topological semimetal materials have been proposed theoretically and realized experimentally. For example, graphene has been demonstrated to be a 2D Dirac semimetal with stable Dirac cones due to the weak SOC of carbon atoms, which are protected by time-reversal symmetry and inversion symmetry.^32^ The Weyl semimetallic features of TaAs, TaP, NbAs and NbPd have been demonstrated theoretically and/or experimentally.^18,33–35^ DNL semimetals have also been proposed in three-dimensional (3D) and two-dimensional (2D) materials, such as Cu_2_Si^36^ and MX (M = Pb, Pt; X = S, Te, Se) monolayers.^37^

Graphene-based nanomeshes (GNMs) with arrays of nano-holes show diverse electronic band structures distinct from pristine graphene. Graphitic carbon nitride, g-C_3_N_4_, has been experimentally synthesized for decades.^38^ Likewise, holey generated C_2_N has also been synthesized by a bottom-up wet-chemical reaction in recent years.^39^ However, most graphitic carbon nitrides are semiconductors with relatively large band gaps, *e.g.*, 2.97 eV for g-C_3_N_4_.^40^ In this contribution, we propose three GNMs doped with nitrogen and oxygen atoms with the stoichiometries of C_10_N_3_, C_9_N_4_ and C_10_O_3_, whose electronic band structures in the proximity of Fermi level feature a 2D honeycomb-kagome lattice. The concurrent Dirac bands and kagome bands lead to Dirac and DNL semimetallic states at the Fermi level, which can be regulated by the doping ratio. The porous configurations of the GNMs facilitate anchoring the TMs to avoid clustering. The adsorption of rhenium (Re) atoms to the voids of C_10_N_3_ (Re@ C_10_N_3_) GNMs leads to quantum anomalous Hall (QAH) states. The GNMs offer a promising platform superior to pristine graphene for regulating the topological states.

## Methods and computational details

First-principles calculations were carried out using density functional theory (DFT) implemented in the Vienna *ab initio* simulation package (VASP).^41,42^ The generalized gradient approximation (GGA) in the formula proposed by Perdew, Burke, and Ernzerhof (PBE) was adopted for the exchange-correlation functional.^43^ The electron–ion interaction was described by the projector-augmented-wave (PAW) potentials.^44^ The Brilloiun zone (BZ) integration was sampled on a grid of 7 × 7 × 1 *k*-points according to the Monkhorst–Pack method for structural optimization. The energy cutoff employed for plane-wave expansion of the electron wavefunction was set to 520 eV. Structural optimizations were carried out using a conjugate gradient (CG) method until the remaining force on each atom was less than 0.01 eV Å^−1^. The supercell was repeated periodically along the *x*- and *y*-directions, while a vacuum space of up to 20 Å was applied along the *z*-direction to exclude the interaction between neighboring images. Maximally localized Wannier functions (MLWF) were constructed using the WANNIER90 and the Wannier Tools packages.^45,46^

## Results and discussion

### A. Tight-binding model for the honeycomb-kagome lattice

**Fig. 1:**
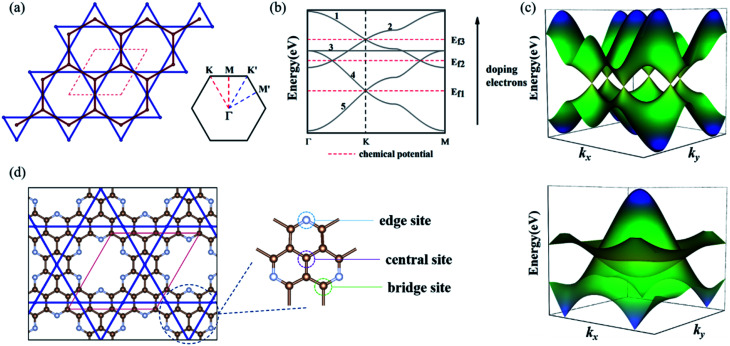
(a) Schematic diagram of the honeycomb-kagome lattice and the first Brillouin zone. The honeycomb lattice is connected by red lines, while the kagome lattice is connected by blue lines. The red dotted line represents the unit cell. (b) The band structure of the honeycomb-kagome lattice based on the TB model. Dirac cone and DNL appear in turn as electrons are doped. (c) The 3D diagram of the Dirac cone and DNL. (d) The structure of a GNM, taking C_10_N_3_ GNM as an example.

We considered a 2D hybrid lattice comprised of a honeycomb and a kagome sublattice, as shown in Fig. 1(a). The unit cell contained five sites with two equivalent basis vectors with an angle of 60° between them. By placing one p_z_ atomic orbital on each site and considering the electron hopping between adjacent sites in each sublattice and the nearest-neighbor hopping between the two sublattices without SOC, the effective tight-binding (TB) Hamiltonian is written as1
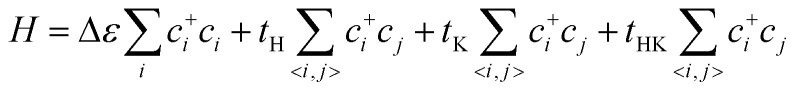
where Δ*ε* is the onsite energy difference between the honeycomb and kagome sublattices, *c*^+^_*i*_/*c*_*i*_ is the creation/annihilation operator of an electron on the *i*-th site, and *t*_H_, *t*_K_, and *t*_HK_ represent the electron-hopping energies within and between the honeycomb and kagome sublattices, respectively, as shown in Fig. S1.† We set these parameters of Δ*ε*, *t*_H_, *t*_K_, and *t*_HK_ as 1.2, −0.3, −0.4, and 0.1 eV, respectively. The diagonalization of the above Hamiltonian generated five electronic bands in the reciprocal space labeled from 1 to 5, as shown in Fig. 1(b). It was interesting to see that two sets of bands derived from the honeycomb and kagome sublattices intersected each other, forming a DNL and two Dirac cones, as shown in Fig. 1(c), which enabled us realize different topological quantum states by regulating the position of the Fermi level (the number of electrons of the system), labeled by *E*_f1_, *E*_f2_, *E*_f3_. The intersection of the Dirac bands (1 and 2) and the kagome bands (3–5) could be adjusted by varying the onsite energy difference between them. Moreover, with the increase in the coupling strength (*t*_HK_), the Dirac cones in the honeycomb bands and kagome bands moved upward and downward, respectively, which regulated the position of the nodal line, as shown in Fig. S2.† When *t*_HK_ exceeded a critical value, the two sets of bands separated and the intersecting nodal line disappeared, but the Dirac cones were preserved. More details about the tight-binding model are presented in the ESI†.

### B. Atomic and electronic structures of the GNMs

To realize the Dirac and DNL states of the honeycomb-kagome lattice, we considered two GNMs with arrays of hexagonal voids, where the edge carbon atoms were replaced by N or O atoms, as shown in Fig. 1(d), yielding the stoichiometries of C_10_N_3_ and C_10_O_3_, respectively. Further substituting the center C atom of C_10_N_3_ with an N atom led to a C_9_N_4_ GNM. Structural optimizations revealed the lattice constants of 9.71, 9.64, and 9.66 Å for the C_10_N_3_, C_9_N_4_, and C_10_O_3_ GNMs. The planar configurations were well preserved in the GNMs, ensuring the sp^2^-hybridization of the p_*z*_ orbitals perpendicular to the basal plane to form a conjugated π orbital throughout the frameworks.

**Fig. 2:**
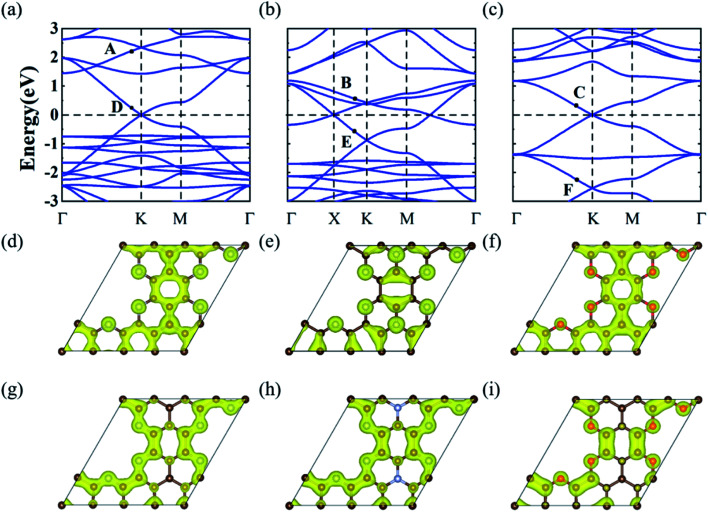
(a)–(c) Band structures of C_10_N_3_, C_9_N_4_ and C_10_O_3_, respectively. (d)–(f) Charge densities of the A, B and C states, respectively, decomposed from Dirac bands. (g)–(i) Charge densities of the D, E, and F states, respectively, decomposed from kagome bands. The isosurface values for (d) to (i) were set at 0.002 e Å^−3^.

The electronic band structures of the three GNMs obtained from the DFT calculations were depicted in Fig. 2(a)–(c). These GNMs showed very similar band structure characteristics, *i.e.*, containing two bands derived from the honeycomb lattice and three bands from the kagome lattice. To reveal the origins of the five special bands, we calculated the spatial distribution of the charge density at the points A–F, which arose from the kagome and Dirac bands, respectively. From Fig. 2(d)–(i), one can find that the states of A, B, and C were mainly contributed by the p_*z*_ orbitals of the central atom and some of the surrounding atoms, while the D, E, and F states stemmed mainly from the p_*z*_ orbitals of the atoms except the central one. The features of the charge density distribution basically accorded with the characteristics of the honeycomb and kagome sublattices. Therefore, these GNMs can be regarded as candidate materials to realize the topological semi-metallicity of the honeycomb-kagome lattice. The orientation-dependent Fermi velocities determined from the gradient of bands near the Fermi level are presented in Table 1, from which we can see that these GMNs have Fermi velocities comparable to graphene (8.45 × 10^5^ m s^−1^),^47^ SiC_3_ (5.91 × 10^5^ m s^−1^),^47^ silicene (5.36 × 10^5^ m s^−1^),^48^ germanene (5.30 × 10^5^ m s^−1^),^48^ and higher than Si_3_C (4.92 × 10^5^ m s^−1^),^47^ 2D MOF [Pb(C_6_H_5_)_3_] (1.72 × 10^5^ m s^−1^),^49^ [Ni_2_C_24_S_6_H_12_] (1.25 × 10^5^ m s^−1^),^50^*etc.* Moreover, the conjugated π orbitals of graphene were largely preserved in these GMMs, which were responsible for the semimetallic features. For g-C_3_N_4_ and C_2_N, the conjugated π orbitals were destroyed due to the high N concentrations, which thus lead to a semiconducting nature.

**Table 1:** The Fermi velocity at the linear intersection at the Fermi level

Fermi velocity (10^5^ m s^−1^)	*Γ* → *K*	*K* → *Γ*	*Γ* → *X*	*X* → *K*
C_10_N_3_	−6.22	6.42	/	/
C_9_N_4_	/	/	−7.87	3.51
C_10_O_3_	−5.42	5.28	/	/

Notably, these GNMs had different positions of Fermi level. This can be attributed to the different numbers of valence electrons of C, N, and O atoms in these GNMs. For C_10_N_3_ GNM, the Fermi level resided right at the Dirac point of the kagome bands. When the center C atoms were replaced by N atoms (C_9_N_4_), two more electrons were added into the unit cell. According to the TB model of the honeycomb-kagome lattice, the Fermi level will be pushed upward to the position of the DNL. As the edge N atoms of C_9_N_4_ GNM were substituted by O atoms (C_10_O_3_), six electrons per unit cell were introduced, and the Fermi level moved upward to the Dirac point of the Dirac bands. Therefore, the Dirac cones at the Fermi level have different origins for the C_10_N_3_ and C_10_O_3_ GNM. This offers a promising strategy to regulate the topological electronic states of GNMs.

### C. Topological properties of Re@C_10_N_3_

**Fig. 3:**
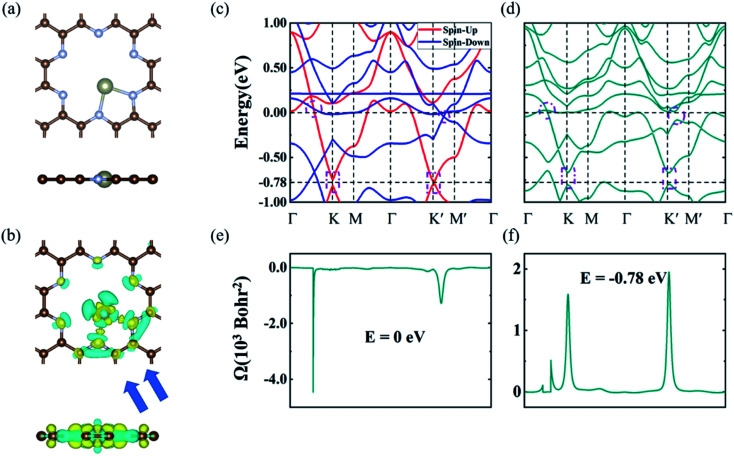
(a) The most stable adsorption configuration of Re@C_10_N_3_. The upper panel is a top view, the lower panel is a side view. (b) The charge density difference of the Re@C_10_N_3_ adsorption configuration; yellow and cyan regions represent electron accumulation and depletion, respectively. The isosurface values for (b) were set at 0.007 e Å^−3^. (c) and (d) the band structures of Re@C_10_N_3_ without and with considering SOC, respectively. The red and blue colored bands without SOC represent the spin-up and spin-down character, respectively. (e) and (f) the Berry curvature at the Fermi level and the energy at −0.78 eV when considering SOC, respectively.

Considering the porous structure and rich adsorption sites of the GNMs, we studied the effects of TM dopants on the electronic and topological properties of the GNMs. We chose Re as the dopant and tested the most stable adsorption configuration and binding energy (*E*_b_) on the GNMs. Wherein, the binding energy can be defined as *E*_b_ = *E*_tot_ − *E*_GNM_ − *E*_TM_, where *E*_tot_, *E*_GNM_ and *E*_TM_ are the energies of TM@GNM, GNM, and TM, respectively.^51,52^ Three initial adsorption sites labeled 1–3 were considered, as shown in Fig. S3.† The results showed that Re tends to stay at site-2 and lies in the holes of C_10_N_3_ and C_9_N_4_, but above the hole of C_10_O_3_, as depicted in Fig. 3(a) (Re@C_10_N_3_) and Fig. S4† (Re@C_9_N_4_ and Re@C_10_O_3_). Bader charge analysis indicated that the electrons transferring from Re to the GNMs were respectively 1.14 |e| (C_10_N_3_), 1.10 |e| (C_9_N_4_), and 0.53 |e| (C_10_O_3_), as listed in Table 2, which were consistent with the different charge density profiles plotted in Fig. 3(b) (Re@C_10_N_3_) and Fig. S5† (Re@C_9_N_4_ and Re@C_10_O_3_). The length of the bonds between the Re and edge atoms were 1.98, 1.94, and 2.16 Å for C_10_N_3_, C_9_N_4_, and C_10_O_3_, respectively. C_10_N_3_ had the strongest binding strength to the Re atom with *E*_b_ = −4.65 eV, whereas C_10_O_3_ had the weakest binding strength of −1.36 eV. Therefore, we focused on the electronic and topological properties of Re@ C_10_N_3_ in the following section.

**Table 2:** The binding energy, charge transfer and bond length between the Re atom and the CNO material. Among these, a negative binding energy means a more stable structure, while a higher charge transfer means a stronger bond between atoms. Besides, the bond length can also indicate the stability of the bond

	*E*_b_ (eV)	Δ*Q*_Re → CNO_ (e^−^)	*L*_Re–N(O)_ (Å)
Re@C_10_N_3_	−4.65	1.14	1.98
Re@C_9_N_4_	−4.54	1.10	1.94
Re@C_10_O_3_	0.78	0.53	2.16

The electronic band structures of Re@C_10_N_3_ without and with SOC were depicted in Fig. 3(c) and (d). The band without SOC was spin-polarized, from which we can see that the Fermi level had moved upward compared with the pristine C_10_N_3_ GNM, which can be attributed to the electron transfer from Re to C_10_N_3_. The Dirac cones moved down to the region of about 0.78 eV below the Fermi level. New band crossings also emerged near the Fermi level. The calculated orbital-resolved band structures, as shown in Fig. S6,† showed that the band crossings near the Fermi level and the Dirac cones −0.78 eV below the Fermi level were mainly contributed by C-p_*z*_, N-p_*z*_, and Re-d orbitals. The contributions of Re atoms would harbor a significantly strong SOC strength. When SOC was involved, the Dirac cones were opened up with a band gap of 120 meV, while the band crossings near the Fermi level were also gapped by 1.47 meV and 88.28 meV, respectively.

Generally, such SOC-induced band evolution is a typical scenario for the existence of topologically nontrivial electronic states. We therefore revealed the topological characteristics by means of the maximally localized Wannier functions (MLWFs) implemented in the WANNIER90 and the WannierTools packages in combination with DFT calculations.^45,46^ According to the origins of these electronic states, we constructed the MLWFs using the d orbitals of the Re atoms and the p_*z*_ orbitals of the C and N atoms. We calculated the *k*-resolved Berry curvature *Ω*(*k*) by using the Kubo formula:^53,54^2
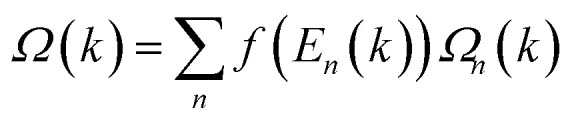
3
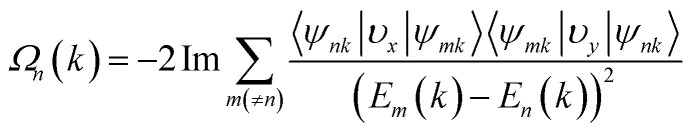
where the summation is over all of the occupied states, *E*_*n*_(*k*) represents the eigenvalue of the Bloch function *ψ*_*nk*_, *f* is the Fermi–Dirac distribution function, and *υ*_*x*_/*υ*_*y*_ are the velocity operators in the *x*/*y* direction. Then, the Chern number (*C*) can be obtained by integrating the Berry curvature over the first Brillouin zone:4
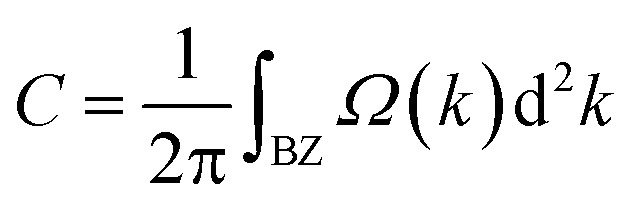


The Chern number can also be viewed as the number of electronic charges pumped across the unit cell in the course of a cycle, which is evaluated from the evolution of the hybrid Wannier charge centers during a time-reversal pumping process. Moreover, the chiral edge states appearing within the band gap can also be evidence of the topological properties.

**Fig. 4:**
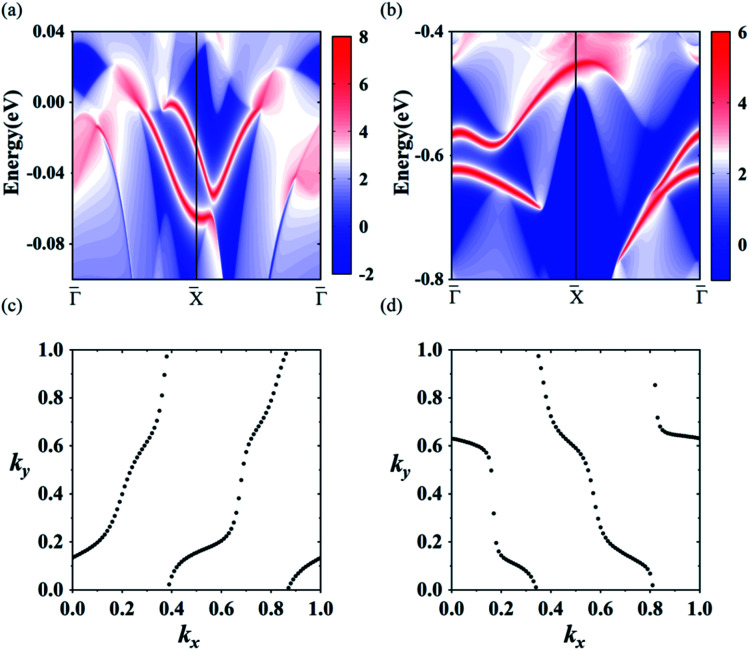
(a) and (b) the edge states along the [010] orientation at the Fermi level and the energy at −0.78 eV, respectively. (c) and (d) the winding number at the two energy levels, respectively.

Using the above strategy, we first checked the topological aspects of the band gap near the Fermi level. From the Berry curvature depicted in Fig. 3(e), one can find a peak appears at every band gap but was almost zero in the other regions. The calculated Chern number for the band was *C* = 2, which was consistent with the winding numbers shown in Fig. 4(c). Accompanied by the nonzero Chern number (*C* = 2), two dissipationless and topologically protected edge states emerged on each side of a Re@C_10_N_3_ ribbon. As shown in Fig. 4(a), the bulk states were connected by two topologically nontrivial edge states with the same orientation, which was consistent with the magnitude of the Chern number. For the band gap 0.78 eV below the Fermi level, the calculated Chern number was *C* = −2, which was in good agreement with the winding numbers shown in Fig. 4(d) and two topologically nontrivial edge bands with opposite directions shown in Fig. 4(b). Therefore, both band gaps were topologically nontrivial, and thus are promising for achieving QAH effects.

## Conclusions

In summary, we proposed a single-orbital TB model of a honeycomb-kagome lattice with a concurrent Dirac cone and DNL. By means of first-principles calculations, we predicted three GNMs (C_10_N_3_, C_9_N_4_, and C_10_O_3_) as candidate materials to realize this TB model. The electronic band structures of these GNMs exhibited similar characteristics of Dirac and DNL semimetals depending on the valence electron numbers of the dopant atoms that regulate the position of the Fermi level. The adsorption of rhenium (Re) atoms to the voids of C_10_N (Re@ C_10_N_3_) GNMs induced QAH states with nonzero Chern numbers and chiral edge states. Beside the topological aspects of TM@GNMs, the active TM atoms anchored onto the GNMs also offer a promising strategy for single-atom catalysis (SAGs). Topological surface states are expected to act as an electron path in catalytic processes and thus may affect the catalytic activity.^55–64^ Our work provides an ideal candidate system for the study of topological catalysis, where the catalytic efficiency is highly correlated to the topological aspects of materials.

## Conflicts of interest

The authors declare no competing financial interest.

## Supplementary Material

NA-004-D2NA00132B-s001

## References

[cit1] Hasan M. Z., Kane C. L. (2010). Rev. Mod. Phys..

[cit2] Schnyder A. P., Ryu S., Furusaki A., Ludwig A. W. (2008). Phys. Rev. B.

[cit3] Qi X.-L., Zhang S.-C. (2011). Rev. Mod. Phys..

[cit4] Li S.-s., Ji W.-x., Hu S.-j., Zhang C.-w., Yan S.-s. (2017). ACS Appl. Mater. Interfaces.

[cit5] Haldane F. D. M. (1988). Phys. Rev. Lett..

[cit6] Liu Z., Zhao G., Liu B., Wang Z., Yang J., Liu F. (2018). Phys. Rev. Lett..

[cit7] Zhang X., Zhao M. (2015). RSC Adv..

[cit8] Zhang S.-j., Zhang C.-w., Zhang S.-f., Ji W.-x., Li P., Wang P.-j., Li S.-s., Yan S.-s. (2017). Phys. Rev. B.

[cit9] Weeks C., Hu J., Alicea J., Franz M., Wu R. (2011). Phys. Rev. X.

[cit10] Hu J., Alicea J., Wu R., Franz M. (2012). Phys. Rev. Lett..

[cit11] Hu J., Zhu Z., Wu R. (2015). Nano Lett..

[cit12] Zhang H., Lazo C., Blügel S., Heinze S., Mokrousov Y. (2012). Phys. Rev. Lett..

[cit13] Zhang K.-C., Li Y.-F., Liu Y., Zhu Y. (2019). J. Appl. Phys..

[cit14] Krasheninnikov A., Lehtinen P., Foster A. S., Pyykkö P., Nieminen R. M. (2009). Phys. Rev. Lett..

[cit15] Yang B., Zhang X., Wang A., Zhao M. (2019). J. Phys.: Condens. Matter.

[cit16] Yang B., Zhang X., Zhao M. (2017). Nanoscale.

[cit17] Wang Z., Sun Y., Chen X.-Q., Franchini C., Xu G., Weng H., Dai X., Fang Z. (2012). Phys. Rev. B.

[cit18] Weng H., Fang C., Fang Z., Bernevig B. A., Dai X. (2015). Phys. Rev. X.

[cit19] Weng H., Liang Y., Xu Q., Yu R., Fang Z., Dai X., Kawazoe Y. (2015). Phys. Rev. B.

[cit20] Yu R., Weng H., Fang Z., Dai X., Hu X. (2015). Phys. Rev. Lett..

[cit21] Kim Y., Wieder B. J., Kane C., Rappe A. M. (2015). Phys. Rev. Lett..

[cit22] Xie L. S., Schoop L. M., Seibel E. M., Gibson Q. D., Xie W., Cava R. J. (2015). APL Mater..

[cit23] Zeng M. , Fang C., Chang G., Chen Y.-A., Hsieh T., Bansil A., Lin H. and Fu L., arXiv:1504.03492, 2015

[cit24] Mullen K., Uchoa B., Glatzhofer D. T. (2015). Phys. Rev. Lett..

[cit25] Li R., Ma H., Cheng X., Wang S., Li D., Zhang Z., Li Y., Chen X.-Q. (2016). Phys. Rev. Lett..

[cit26] Zdanowicz W., Zdanowicz L. (1975). Annu. Rev. Mater. Sci..

[cit27] Abrikosov A. (1998). Phys. Rev. B.

[cit28] Zhang W., Yu R., Feng W., Yao Y., Weng H., Dai X., Fang Z. (2011). Phys. Rev. Lett..

[cit29] Liu C.-X., Zhang H., Yan B., Qi X.-L., Frauenheim T., Dai X., Fang Z., Zhang S.-C. (2010). Phys. Rev. B.

[cit30] Röber E., Hackstein K., Coufal H., Sotier S. (1979). Phys. Status Solidi B.

[cit31] Koshino M., Ando T. (2010). Phys. Rev. B.

[cit32] Novoselov K. S., Geim A. K., Morozov S. V., Jiang D.-e., Zhang Y., Dubonos S. V., Grigorieva I. V., Firsov A. A. (2004). Science.

[cit33] Lv B., Weng H., Fu B., Wang X. P., Miao H., Ma J., Richard P., Huang X., Zhao L., Chen G. (2015). Phys. Rev. X.

[cit34] Xu S.-Y., Belopolski I., Sanchez D. S., Zhang C., Chang G., Guo C., Bian G., Yuan Z., Lu H., Chang T.-R. (2015). Sci. Adv..

[cit35] Huang S.-M., Xu S.-Y., Belopolski I., Lee C.-C., Chang G., Wang B., Alidoust N., Bian G., Neupane M., Zhang C. (2015). Nat. Commun..

[cit36] Feng B., Fu B., Kasamatsu S., Ito S., Cheng P., Liu C.-C., Feng Y., Wu S., Mahatha S. K., Sheverdyaeva P. (2017). Nat. Commun..

[cit37] Jin Y.-J., Wang R., Zhao J.-Z., Du Y.-P., Zheng C.-D., Gan L.-Y., Liu J.-F., Xu H., Tong S. (2017). Nanoscale.

[cit38] Thomas A., Fischer A., Goettmann F., Antonietti M., Müller J.-O., Schlögl R., Carlsson J. M. (2008). J. Mater. Chem..

[cit39] Mahmood J., Lee E. K., Jung M., Shin D., Jeon I.-Y., Jung S.-M., Choi H.-J., Seo J.-M., Bae S.-Y., Sohn S.-D. (2015). Nat. Commun..

[cit40] Niu P., Zhang L., Liu G., Cheng H. M. (2012). Adv. Funct. Mater..

[cit41] Kresse G., Furthmüller J. (1996). Phys. Rev. B.

[cit42] Kresse G., Hafner J. (1993). Phys. Rev. B.

[cit43] Perdew J. P., Burke K., Ernzerhof M. (1996). Phys. Rev. Lett..

[cit44] Kresse G., Joubert D. (1999). Phys. Rev. B.

[cit45] Mostofi A. A., Yates J. R., Lee Y.-S., Souza I., Vanderbilt D., Marzari N. (2008). Comput. Phys. Commun..

[cit46] Wu Q., Zhang S., Song H.-F., Troyer M., Soluyanov A. A. (2018). Comput. Phys. Commun..

[cit47] Zhao M., Zhang R. (2014). Phys. Rev. B.

[cit48] Cahangirov S., Topsakal M., Aktürk E., Şahin H., Ciraci S. (2009). Phys. Rev. Lett..

[cit49] Wang Z., Liu Z., Liu F. (2013). Nat. Commun..

[cit50] Wei L., Zhang X., Zhao M. (2016). Phys. Chem. Chem. Phys..

[cit51] Choi C., Back S., Kim N.-Y., Lim J., Kim Y.-H., Jung Y. (2018). ACS Catal..

[cit52] Ling C., Shi L., Ouyang Y., Zeng X. C., Wang J. (2017). Nano Lett..

[cit53] Yao Y., Kleinman L., MacDonald A., Sinova J., Jungwirth T., Wang D.-s., Wang E., Niu Q. (2004). Phys. Rev. Lett..

[cit54] Yao Y., Fang Z. (2005). Phys. Rev. Lett..

[cit55] Xiao J., Kou L., Yam C.-Y., Frauenheim T., Yan B. (2015). ACS Catal..

[cit56] Li L., Zeng J., Qin W., Cui P., Zhang Z. (2019). Nano Energy.

[cit57] Kong X.-P., Jiang T., Gao J., Shi X., Shao J., Yuan Y., Qiu H.-J., Zhao W. (2021). J. Phys. Chem. Lett..

[cit58] Boukhvalov D. W., Kuo C.-N., Nappini S., Marchionni A., D'Olimpio G., Filippi J., Mauri S., Torelli P., Lue C. S., Vizza F. (2021). ACS Catal..

[cit59] Yang Q., Le C., Li G., Heine T., Felser C., Sun Y. (2021). Appl. Mater. Today.

[cit60] Yang Q., Li G., Manna K., Fan F., Felser C., Sun Y. (2020). Adv. Mater..

[cit61] Li G., Fu C., Shi W., Jiao L., Wu J., Yang Q., Saha R., Kamminga M. E., Srivastava A. K., Liu E. (2019). Angew. Chem..

[cit62] Li G., Xu Q., Shi W., Fu C., Jiao L., Kamminga M. E., Yu M., Tüysüz H., Kumar N., Süβ V. (2019). Sci. Adv..

[cit63] Rajamathi C. R., Gupta U., Kumar N., Yang H., Sun Y., Süβ V., Shekhar C., Schmidt M., Blumtritt H., Werner P. (2017). Adv. Mater..

[cit64] Chen H., Zhu W., Xiao D., Zhang Z. (2011). Phys. Rev. Lett..

