# Supplementary material for: Tunable topological electronic states in the honeycomb-kagome lattices of nitrogen/oxygen-doped graphene nanomeshes
Source: Nanoscale Adv. 2022 Apr 6;4(9):2201–7. doi: 10.1039/d2na00132b (PMC9419200; doi:10.1039/d2na00132b)
Supplement: NA-004-D2NA00132B-s001 [file NA-004-D2NA00132B-s001.pdf]

Electronic Supplementary Material (ESI) for Nanoscale Advances.
This journal is © The Royal Society of Chemistry 2022

Supplementary material for

Tunable topological electronic states in the Honeycomb-kagome lattices of nitrogen/oxygen-doped graphene nanomeshes

Yiming Lu, Xuejia Fan, Xikui Ma*, Jian Liu, Yangyang Li, Mingwen Zhao*

School of Physics, Shandong University, Jinan, Shandong, 250100, China

[*] Corresponding authors: Email: mxk2022@sdu.edu.cn (XM); zmw@sdu.edu.cn (MZ)

TB-modal

Here, we investigate the influence of the onsite energy difference between the two sublattices ($\Delta\varepsilon$), and the hopping energy (t_H, t_K, t_{HK}) on bands, especially t_{HK}, which represents the coupling strength between the two sublattices, as shown in Fig. S1. Firstly, $\Delta\varepsilon$ affects the relative position between the two sets of bands. Secondly, the hopping energy within the two sublattices affect the spread of the two sets of bands on the energy scale. Next, we focus on exploring the influence of coupling strength on the bands.

The Hamiltonian matrix is given below, where the parameter a represents half of the lattice constants whose value is meaningless but affects the hopping energy. We fix the onsite energy difference between the two sublattices to 1.2 eV and the hopping energy within honeycomb and kagome sublattices to -0.3 eV and -0.4 eV, respectively. Fig. S2 shows the trend of bands with parameter t_{HK} varying from 0 eV to 0.9 eV, where the red line in bold corresponds to the case when t_{HK} is 0 eV. It is clear that one of the Dirac cone belonging to the kagome sublattice moves down and the another belonging to the honeycomb sublattice moves up with the coupling strength increasing, and there is a critical value (0.45 eV) where the two sets of band are separated with no intersection.

$$H = \begin{bmatrix} H_{11} & H_{12} & H_{13} & H_{14} & H_{15} \\ H_{21} & H_{22} & H_{23} & H_{24} & H_{25} \\ H_{31} & H_{32} & H_{33} & H_{34} & H_{35} \\ H_{41} & H_{42} & H_{43} & H_{44} & H_{45} \\ H_{51} & H_{52} & H_{53} & H_{54} & H_{55} \end{bmatrix}$$, in which

$$H_{11} = \Delta\varepsilon;$$

$$H_{22} = \Delta\varepsilon;$$

$$H_{33} = 0;$$

$$H_{44} = 0;$$

$$H_{55} = 0;$$

$$H_{12} = t_H \left[\exp\left(\frac{2\sqrt{3}}{3} \mathrm{i} k_x a \right) + \exp\left(-\frac{\sqrt{3}}{3} \mathrm{i} k_x a + \mathrm{i} k_y a \right) + \exp\left(-\frac{\sqrt{3}}{3} \mathrm{i} k_x a - \mathrm{i} k_y a \right) \right];$$

$$H_{34} = 2t_K \cos\left(k_y a\right);$$

$$H_{35} = 2t_K \cos\left(\frac{\sqrt{3}}{2} k_x a - \frac{k_y a}{2} \right);$$

$$H_{45} = 2t_K \cos\left(\frac{\sqrt{3}}{2} k_x a + \frac{k_y a}{2} \right);$$

$$H_{13} = t_{HK} \exp\left(-\frac{\sqrt{3}}{6} \mathrm{i} k_x a + \frac{\mathrm{i} k_y a}{2} \right);$$

$$H_{14} = t_{HK} \exp\left(-\frac{\sqrt{3}}{6} \mathrm{i} k_x a - \frac{\mathrm{i} k_y a}{2} \right);$$

$$H_{15} = t_{HK} \exp\left(\frac{\sqrt{3}}{3} \mathrm{i} k_x a \right);$$

$$H_{23} = t_{HK} \exp\left(\frac{\sqrt{3}}{6} \mathrm{i} k_x a - \frac{\mathrm{i} k_y a}{2} \right);$$

$$H_{24} = t_{HK} \exp\left(\frac{\sqrt{3}}{6} \mathrm{i} k_x a + \frac{\mathrm{i} k_y a}{2} \right);$$

$$H_{25} = t_{HK} \exp\left(-\frac{\sqrt{3}}{3} \mathrm{i} k_x a \right).$$

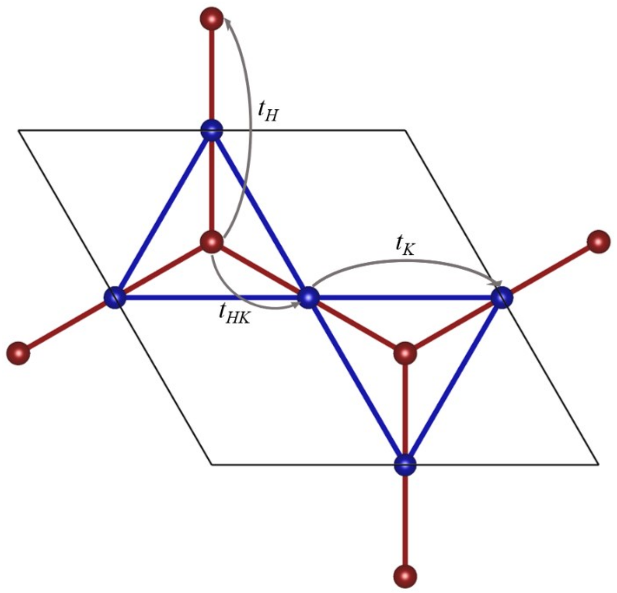

Fig. S1 The HK lattice and hopping energy within the two sublattice and between them.

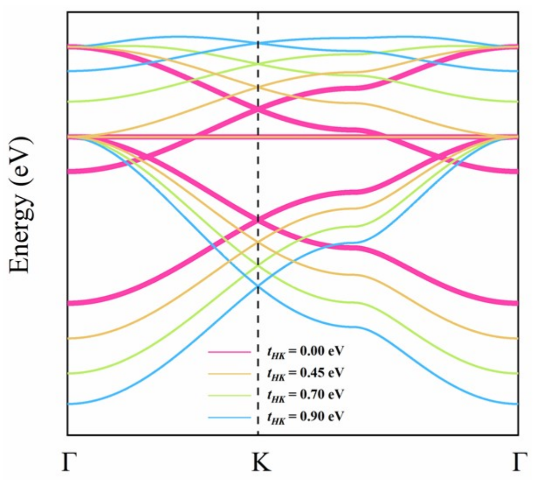

Fig. S2 The trend of band with parameter t_{HK} varying from 0 eV to 0.9 eV. The red line in bold corresponds to the situation when hopping energy is 0 eV.

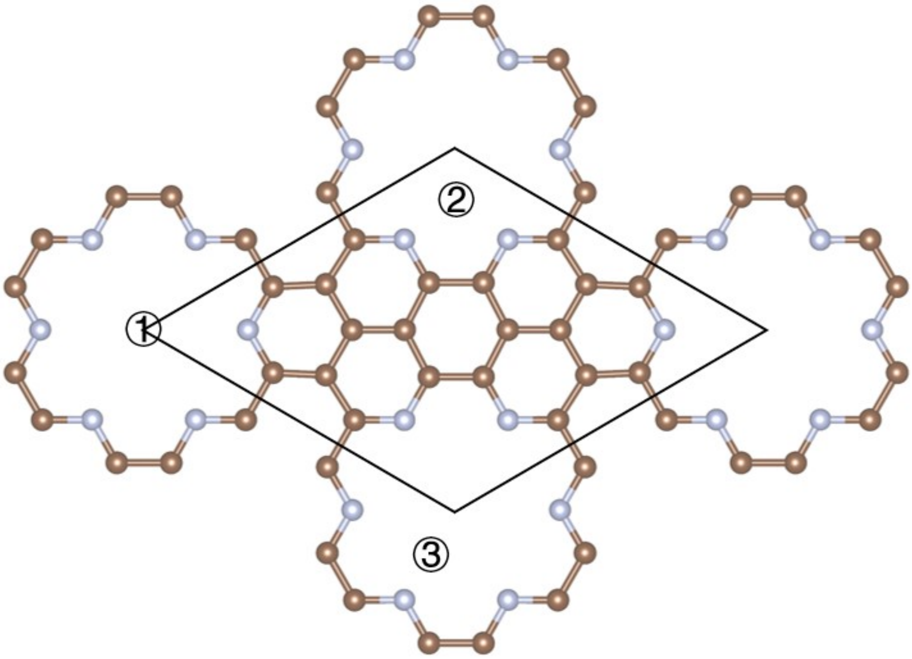

Fig. S3 Three initial adsorption sites were considered, labeled 1, 2, 3, respectively.

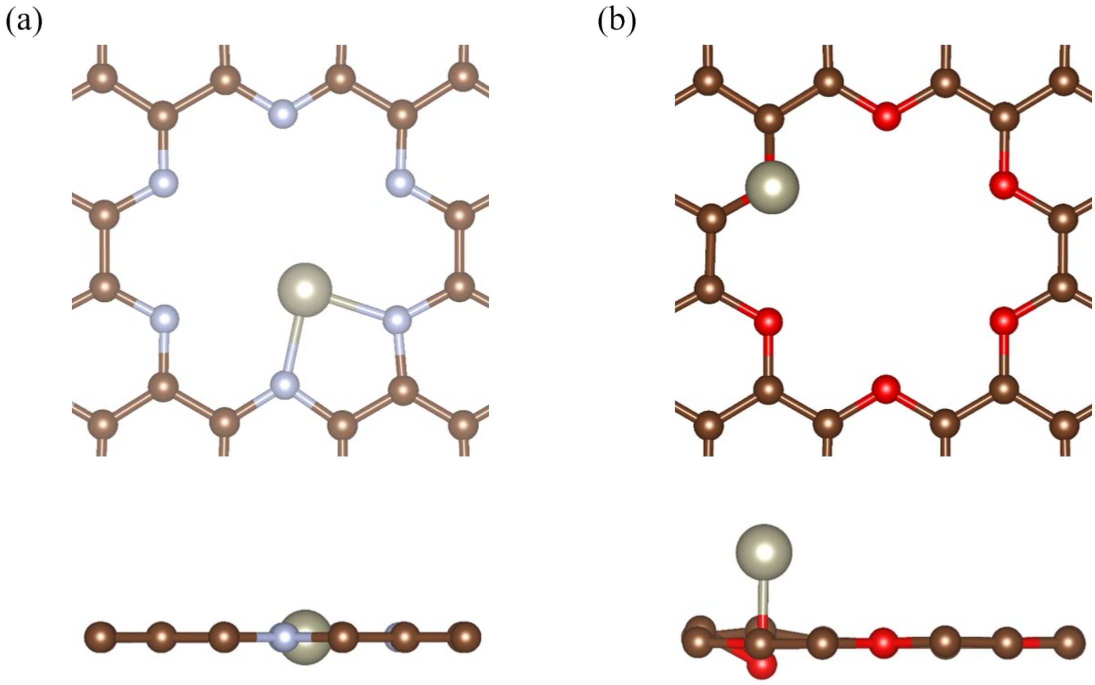

Fig. S4 The most stable adsorption configuration of Re@C_9N_4 and Re@$C_{10}O_3$. The upper panel is a top view, the lower panel is a side view.

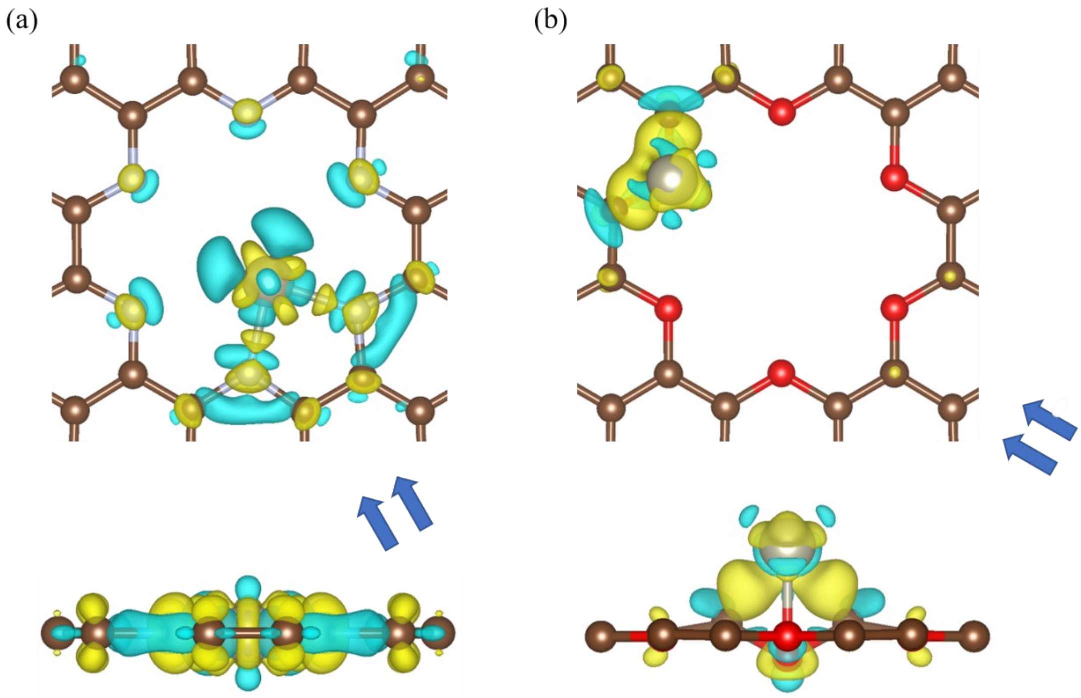

Fig. S5 The charge density difference of the Re@C_9N_4 and Re@$C_{10}O_3$ adsorption configuration. The isosurface values for (a) and (b) are set to 0.007 e Å^{-3}.

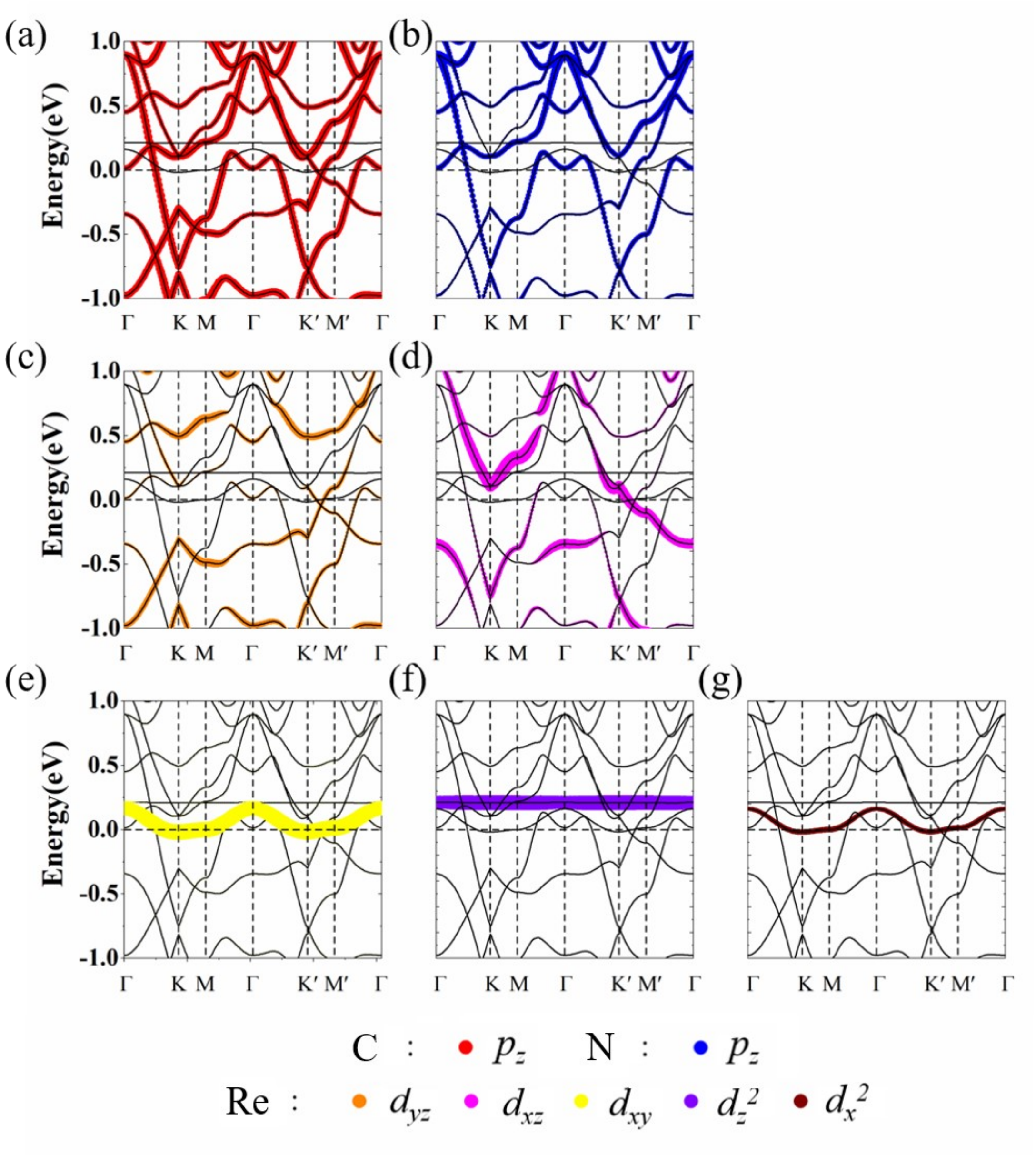

Fig. S6 The calculated orbital-resolved band structure for $Re@C_{10}N_3$ without SOC.
